# Growing dune encroachment threatens the habitability of the western Nile riverbank

**DOI:** 10.1038/s41598-026-35048-w

**Published:** 2026-01-26

**Authors:** Maysa M. N. Taha, Essam Heggy, R. R. Ali, Mahmoud Abd-Elhameed, Sara S. Fouad, Mohamed Ramah

**Affiliations:** 1 Department of Geology, Faculty of Science, Capital University, Cairo, Egypt; 2 Institut de Physique du Globe de Paris, Université Paris Cité, CNRS, Paris, France; 3 Viterbi School of Engineering, University of Southern California, Los Angeles, CA USA; 4 Soils and Water Use Department, National Research Centre, Cairo, Egypt; 5 School of Engineering and Design, Technical University of Munich, Munich, Germany; 6 Earth and Life Institute, Université Catholique de Louvain, Louvain La Neuve, Belgium; 7 Geophysical Sciences Department, National Research Centre, Cairo, Egypt

**Keywords:** Dune encroachment, Sand stabilization, Agricultural sustainability, Change in land use, Deserts, Nile riverbank, Climate sciences, Ecology, Ecology, Environmental sciences, Natural hazards

## Abstract

Sand dune encroachment poses a significant environmental challenge for peri-urban and rural communities in the North African desert, which is home to more than one-third of the region’s population. The continuous movement of sand dunes disrupts residential development, infrastructure, and agricultural systems, threatening food and energy supplies in regions already sensitive to climate variability. The subsequent decline in habitability in such areas often leads to external migration, which triggers heightened socioeconomic and geopolitical instability. As part of the North African Sahara, the West El-Minya Governorate in Egypt is a crucial case study for Saharan areas where growing dune encroachment compromises extensive and critical agricultural developments. We investigate and quantify the primary drivers of sand movement, including wind speed and direction, surface elevation, slope, land use, vegetation cover, and soil cohesion, through the Sand Dune Encroachment Vulnerability Index. Our results reveal that agricultural soils with inadequate irrigation, particularly those adjacent to bare lands, are most susceptible to encroachment. Furthermore, 14% of the total cultivated area is affected by dune encroachment, resulting in estimated annual economic losses of $263 million. Moreover, ~42% of newly established agricultural lands are situated in zones of very high vulnerability, with anticipated productivity reductions of 25% and annual rehabilitation costs approximately $52 million. Transport infrastructures are also impacted, with key highways incurring $6.5 million annually in sand clearance due to recurring dune interference. The proximity of dune-encroached areas to irrigation canals escalates sedimentation rates, deteriorating water quality and incurring additional dredging expenses of $31.3 million per year, with adverse repercussions for agriculture and fisheries. Our study reveals growing dune encroachment, highlighting the urgent need for targeted, nature-based dune stabilization interventions, such as dune leveling and reclamation, in peri-urban Saharan regions. These measures are crucial for preventing further land degradation, reducing population displacement and regional conflict risks, and maintaining the habitability of arid areas.

## Introduction

Desertification is a multifaceted process of land degradation in arid, semi-arid, and dry sub-humid areas, resulting from various drivers, including climatic and anthropogenic changes. It encompasses a wide range of environmental challenges, including soil erosion and degradation, water scarcity, salinization, and vegetation loss^1,2^. Within this broad context, sand dune encroachment emerges as one of the most significant challenges to both urban and agricultural areas on the African continent. It has been estimated that 319 million hectares (ha), mostly surrounding the Sahara, are vulnerable to specific desertification hazards driven by sand movement^2^.

​​The report by the Food and Agriculture Organization (FAO) and the United Nations Environment Program (UNEP) on the assessment of land degradation in Africa suggests that large areas of countries in the northern part of the continent suffer from serious desertification challenges^2^. While desertification manifests in various forms, dune migration is particularly challenging in the semi-arid parts of the West African Sahara due to its rapid movement at a rate of up to a few kilometers per year, which encroaches on urban and agricultural areas. Wind erosion and human activities, such as overgrazing, deforestation, and improper watering of crops, are all contributing factors to this phenomenon^3^. Moreover, sand dunes are progressively encroaching on farmlands, posing a serious threat to regional agricultural productivity and land stability. This affects vast areas, especially in Egypt, Libya, Tunisia, Algeria, and Morocco, which together account for about a third of the population of this region.

**Fig. 1:**
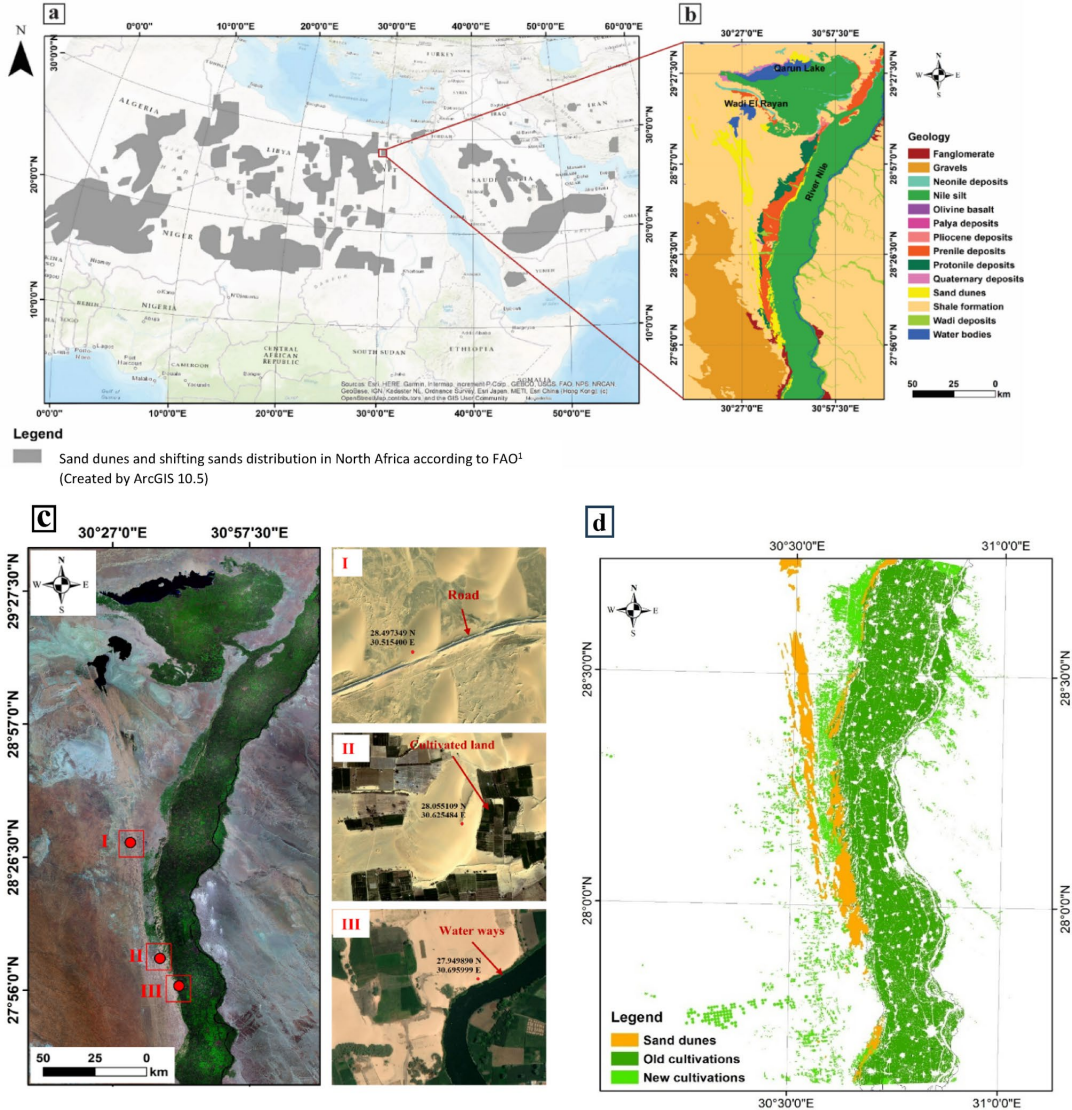
Contextual maps of the study area. (**a**) Distribution of sand dunes in North Africa, according to the FAO^1^ (Created by using ArcGIS 10.5). (**b**) Location and geological units of the study area (after CONCO^9^, Created by using ArcGIS 10.5). (**c**) Examples of sand dune encroachment affecting roads, cultivation, and waterways in the west Al-Minya region (Created by using ArcGIS 10.5). (**d**) The cultivated area is affected by encroachment from sand dunes (ESA CCI Land Cover 2016, Processed by using ArcGIS 10.5).

Several hydroclimatic and meteorological factors drive the expansion of sand fields, affecting dune dynamics and encroachment in the vicinity of peri-urban areas The Sahara is expected to undergo substantial transformations in the dynamics of dune fields by the end of the 21 st century, primarily due to changes in wind patterns driven by the global warming phenomenon^5^. The impacts of such an increase in sand dynamics on dune encroachment in agricultural areas of North Africa can be profound, affecting food sufficiency, socio-economic conditions, and geopolitical tensions due to population migration^4,5^. Although most studies focused on the localized impacts of sand encroachment on small rural communities, the large-scale impacts on densely populated areas were poorly addressed, as they have only recently been observed. In this study, we address the above deficiency. As part of North Africa, the highly populous Egypt faces significant environmental challenges caused by sand encroachment, which occurs over extensive areas (Fig. 1), mainly due to vast desert areas bordering the narrow strip of arable land along the Nile Riverbank, making it highly susceptible to desertification^6,7^. Sand encroachment phenomena are widespread in Egypt, causing ~ 20% of its total area to be covered with aeolian windblown deposits and sand dunes, which threaten productive agricultural land^6^. The total agricultural area affected by this phenomenon is estimated at approximately 762,000 ha out of the country’s total 3.8 million ha of agricultural land^8^. The economic losses are estimated to be ~ 25% of the average annual agricultural land productivity^3,7^. Sustainable agricultural development is pivotal for conserving and enhancing natural resources to address the escalating global demand for farming products, ensuring their quantity and quality for future generations^10^.

Egypt has a predominantly arid to hyper-arid climate, and only ~ 4% of its land surface is currently habitable, while 96% is harsh desert^11–14^. As such, Egypt’s increasing food demand, driven by the rapid population growth, requires an urgent expansion of agricultural areas^14,15^. To address this requirement, the nation has initiated several extensive land reclamation endeavors. Notably, the El-Salam Canal, situated west of the Suez Canal, and the El-Sheikh Jaber Canal, located east of the Suez Canal, collectively referred to as the Sinai Project, seek to reclaim approximately ~251,000 ha^7^. Another significant endeavor is the El-Sheikh Zayed Canal, which aims to reclaim approximately 202,000 ha in the southern New Valley, commonly referred to as the Toshka Project. More recently, a substantial project aims to reclaim 607,000 hectares, with a focus on impoverished regions in Upper Egypt and the Western Desert^7^. Specifically, 169,968 ha are planned for development in the West El-Minya governorate, while 46,944 ha are allocated to Farafra within the Wadi El-Gedid governorate of the Western Desert. The realization of these ambitious reclamation objectives hinges not only on securing water resources^7^ but also on the capacity to mitigate dune encroachment.

To support this objective, our study aims to develop a spatially explicit assessment of sand dune encroachment risk in West El-Minya governorate, an area of promising agricultural development, by applying the Sand Dune Encroachment Vulnerability Index (SDEVI)^16^ using integrated remote sensing and GIS datasets.

The study first quantifies the key land use, meteorological, and geomorphological drivers governing dune mobility in the region. Secondly, it evaluates the implications of dune encroachment on ongoing and planned agricultural development projects. It employs a preliminary quantitative economic analysis framework using available datasets from the literature and governmental reports. Finally, it suggests mitigation strategies at a landscape scale. Our results suggest that areas characterized by high dune mobility, low vegetation cover, and weak soil cohesion exhibit significantly greater vulnerability to sand encroachment, particularly within newly reclaimed agricultural zones.

The findings from this study are expected to support decision-makers in designing targeted mitigation strategies and enhancing the long-term sustainability of land development initiatives in West El-Minya and the region.

### Study area

The narrow stretch of agricultural land along the Western Nile riverbank in Egypt is characterized by its proximity to wind-blown sand. This geospatial setup has seen increasing scientific and public interest due to the persistent threat of dune encroachment on local farmlands and built-up areas. Notably, in West El-Minya governorate, the southern barchans dunes in this region are migrating and endangering adjacent agricultural lands^17^.

West El-Minya is widely regarded as one of the most promising areas for agricultural development, which will help alleviate food stress in populous Egypt amid growing threats of land degradation and water budget deficits^18^. As such, Egyptian authorities have launched vast reclamation efforts over the past decade, primarily utilizing fossil groundwater from fractured limestone aquifers^19,20^. Furthermore, this region serves as a critical case study for investigating sand dune dynamics due to its ongoing reclamation and sustainable development initiatives^20–24^. It exemplifies the broader challenges faced by Saharan regions across North Africa, where sustaining agricultural productivity, preserving cultural heritage, and supporting rural livelihoods are increasingly threatened by sand encroachment.

Our study area is situated between latitudes 27°56’00” to 29°27’30"N and longitudes 30°27’00” to 30°57’30"E, encompassing 24,179 km² (~2.4 million ha) (Fig. 1). This region exhibits distinct geomorphological features, encompassing tablelands, isolated hills, and three distinct floodplains: the silt plain, the sandy plain, and the gravel plain. Additionally, it features a sand dune belt that extends from the southern Qattara Depression to the west of El-Minya governorate^20–24^. Moreover, it is situated in an intermediate area between productive agricultural areas along the Nile River and the desert (Fig. 1). As such, the region is highly vulnerable to dune encroachment^25^. In many parts of North Africa, the contrast between fertile soils and expanding deserts reflects the continent’s broader environmental issues^26^.

Geologically, the West El-Minya area exhibits diverse geological outcrops. The study area is primarily covered by Middle Eocene carbonate rocks of the Samalut Formation, which are mainly composed of limeston^22,23^. Oligocene/Pleistocene deposits, including sand, gravel, and Oligocene basaltic extrusions, cover the lowland areas^24^. Additionally, the study area encompasses Pliocene deposits of dark clays. The Quaternary alluvial deposits consist of Nile silt, pre-Nile and proto-Nile deposits, sand dunes, wadi and playa deposits, fanglomerate, and gravel^20–24^, as illustrated in Fig. 1. Structurally, the study area is affected by Normal faults with a NW-SE trend, displaying throws between 35 and 50 m to the east^27^.

## Methodology

Sand dunes’ morphology develops from wind erosion processes, and deposition on the land surface^28^. The formation and evolution of these dunes are primarily driven by the movement of windblown sand^29^. In addition, the movement of sand dunes is shaped by several landscape features, including vegetation cover, land use, landform, and wind patterns. Previous studies provide valuable insights into sand dune formation and development, with a primary focus on areas with simple terrain^30,31^. However, sand dune formations also develop in more complex landscapes.

For a comprehensive understanding of dune movement and its impact on the surrounding environment, factors such as wind speed and direction, the availability of sand, and the shape of the dunes themselves must be studied^32,33^. In our study, we utilized the Sand Dune Encroachment Vulnerability Index (SDEVI), developed by Gómez et al.^16^, to assess the vulnerability of the study area to dune encroachment. This index combines several key factors, including land shape, slope, wind conditions, geology, land use, vegetation, and soil moisture, as detected by satellite.

The SDEVI investigates seven key elements: the wind’s speed, the wind’s direction, the surface elevation, the surface slope of the land, land use, the vegetation cover, and soil cohesion. Each factor is given a score from 1 (low risk) to 5 (high risk). These scores are then combined to give an overall vulnerability score for each area. This approach enables us to identify which parts of the landscape are most vulnerable to advancing sand dunes, even in complex terrains.

1 $${\rm SDEVI = SpeFa+DirFa+ EleFa + SloFa + LanFa + VegFa +SoiFa}$$

Where, SpeFa is the Wind Speed Factor; DirFa is the Wind Direction Factor; EleFa is the Surface Elevation Factor; SloFa is the Slope Factor; LanFa is the Land Use Factor; VegFa is the Vegetation Cover Factor; SoiFa is the Soil Cohesion Factor. Each factor was classified on a scale from 1 (very low vulnerability) to 5 (very high vulnerability), following Gómez et al.^16^.

This study utilizes the Map Algebra function in GIS software (ArcMap 10.5) to apply the above formula. Parameters were resampled to the highest resolution available, 20 m by 20 m per pixel, using the nearest neighbor technique. This resolution was based on the land cover map provided by the European Space Agency (ESA) of the Africa 2016 map.

Remote sensing (RS) and Geographic Information System (GIS) tools have been utilized to study the sand dune dynamics, overcoming the limitations of field measurements, which are often time-consuming, costly, and limited to specific dune fields. These technologies assist planners and decision-makers in organizing data, making informed decisions, and facilitating access to extensive spatial datasets^43,44^. The SDEVI provides a valuable tool for evaluating susceptibility to sand dune encroachment, aiding in the implementation of preventive measures and mitigating impacts on communities and infrastructure^16,34^.

### Remote sensing and reanalysis datasets

To ensure full reproducibility of the analysis, all datasets, sensors, and spatial resolutions used in constructing the SDEVI are described below:

Wind Speed (SpeFa): Wind speed data were obtained from the Global Land Data Assimilation System (GLDAS) dataset through NASA’s Giovanni platform^35^. GLDAS provides meteorological variables at a 0.25° spatial resolution, making it suitable for large-scale wind analysis in arid regions.

Wind Direction (DirFa): Wind direction was extracted from the Modern-Era Retrospective Analysis for Research and Applications (MERRA) reanalysis dataset. MERRA supplies monthly wind components at 1.25° resolution, enabling identification of dominant dune-driving wind patterns. (Product: MERRA 3D IAU Tendency, Wind Components V5.2)

Soil Moisture (SoiFa): Soil moisture values were derived from the ESA Climate Change Initiative (CCI) SM v03.2, which combines data from satellite radiometers and radar sensors (e.g., ASCAT, AMSR-E). The dataset has a spatial resolution of 0.25° and provides long-term soil information important for understanding sand mobility and cohesion.

Vegetation (VegFa): Vegetation density was extracted from the ESA CCI Land Cover Africa 2016 dataset. The categories were reclassified based on vegetation abundance:

(1) trees/open water; (2) grassland/cropland; (3) aquatic vegetation; (4) sparse or shrub vegetation; (5) bare soil/urban areas.

Land Use (LanFa): Land use classes were obtained from ESA CCI Land Cover 2016 and reclassified according to vulnerability: (1) bare areas; (2) vegetated surfaces; (3) water bodies; (4) cropland and transportation corridors; (5) urban and built-up areas.

Elevation (EleFa): The elevation factor was derived from the Shuttle Radar Topography Mission (SRTM) Digital Elevation Model (DEM) at 30-m spatial resolution, allowing accurate extraction of dune crest elevations.

Surface Slope (SloFa): Slope was computed from the same SRTM DEM (30 m). Higher slope values were associated with increased vulnerability to dune instability and downslope movement.

Agricultural land extent was extracted from Landsat-8 OLI multispectral imagery with a spatial resolution of 30 m, which enabled accurate delineation of cultivated fields within the study area.

### Geographical information system analysis

All datasets were resampled to a unified spatial grid of 20 × 20 m, based on the highest-resolution available dataset. The Map Algebra tool in ArcGIS 10.5 was used to compute the SDEVI raster layer by summing the reclassified factors according to Eq. (1). The final SDEVI output produced a landscape-level vulnerability map identifying locations at the highest risk of dune encroachment.

### Economic assessment

Due to the lack of economic data sets related to the impact of sand dunes in Egypt, this study employs a preliminary quantitative economic analysis framework using available datasets from the literature and governmental reports to evaluate the costs and benefits associated with sand encroachment and its management in agricultural and infrastructural settings. First, we determine the average annual cost of sand encroachment to farmers per hectare, using the reported figure of approximately $8,011 per hectar as a starting point. This loss shows how sand encroachment directly reduce agriculture land and associated crop yields. Furthermore, the costs associated with maintenance interventions are analyzed, with a particular emphasis on the expenses incurred from removing sand that has accumulated due to sandstorms. Two critical aspects of infrastructure are scrutinized: drainage systems and roads. The estimated cost of clearing these systems is approximately $180 per meter, while the cost of clearing roads is approximately $15 per meter^36^. These figures enable the estimation of the annual cost of maintaining the drainage and roads impacted by sand encroachment^36^.

The analysis also includes the one-time cost of leveling and reclaiming land that has been covered in sand dunes, estimated at $9,523 per hectar^37^. This investment aims to stabilize the affected areas, which will enable the cultivable land area to expand by approximately ~ 32,842 hectars, and is expected to increase annual agricultural revenue by around ~ $263 million^37^. The reported movement rate of sand dunes, at 4.4 m per year^38^ and the associated dust storms, helps us estimate how far encroachment will spread each year and how much land and infrastructure will be affected. The method uses these data points to figure out (1) the annual economic losses from lower agricultural productivity due to encroachment; (2) the costs of clearing infrastructure on a regular basis; (3) the total investment needed for reclamation; and (4) the expected extra income after reclamation. The study employs comparative analysis to determine the cost-effectiveness of reclamation in comparison to ongoing maintenance and loss costs. It also looks at payback periods and long-term economic benefits.

## Results

Our results pertain to constraining the uncertainties in the drivers of sand dune dynamics in West El-Minya, the implications of growing sand encroachment, and evaluating the cost of nature-based mitigation.

### Drivers of sand Dune dynamics

#### Wind speed

**Fig. 2:**
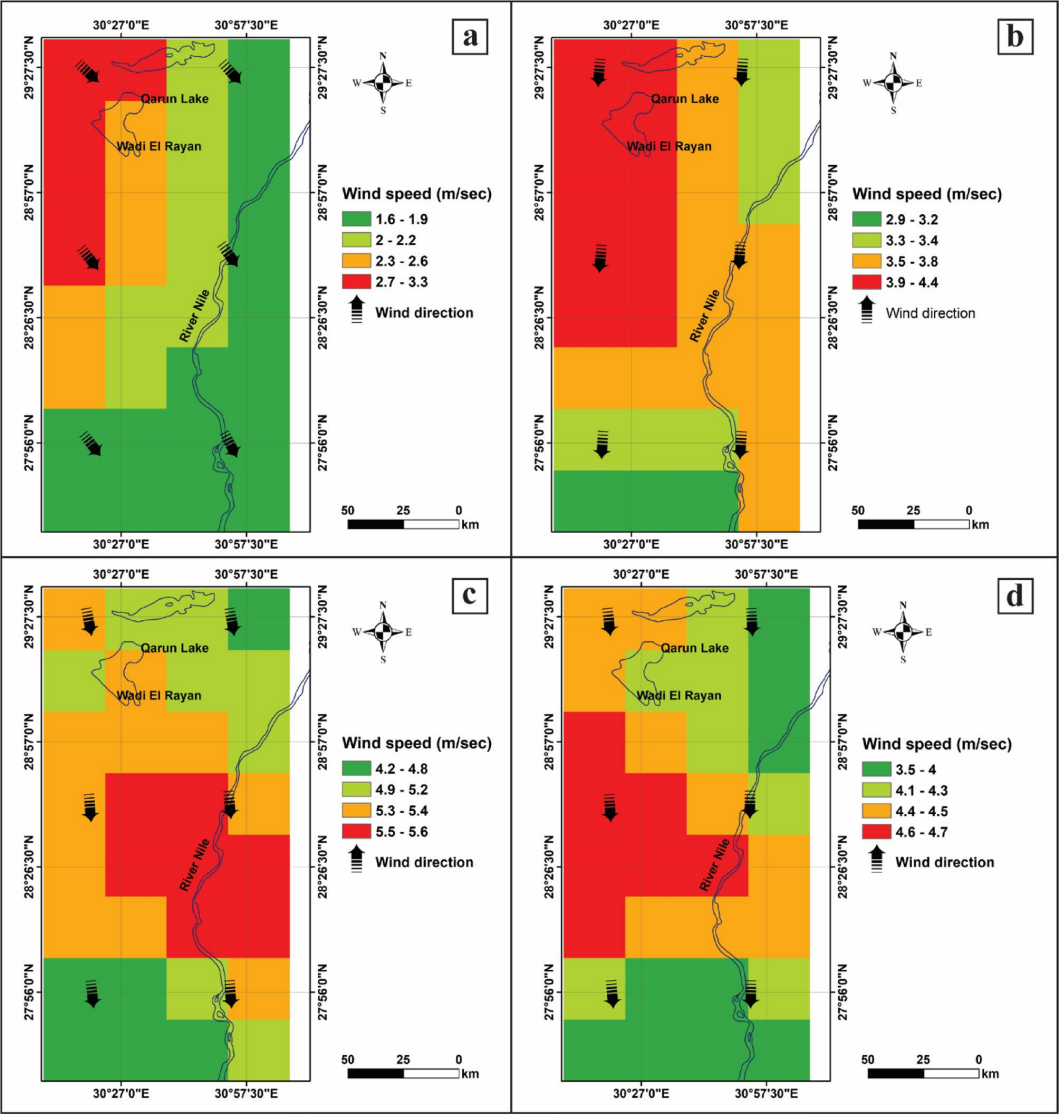
Wind speed (GLDAS, https://giovanni.gsfc.nasa.gov/giovanni/*)* and direction (MERRA 3D IAU Tendency, Wind Components V5.2) in West El-Minya by season (Processed by using ArcGIS 10.5) : (**a**) winter, (**b**) spring, (**c**) summer, and (**d**) autumn.

**Fig. 3:**
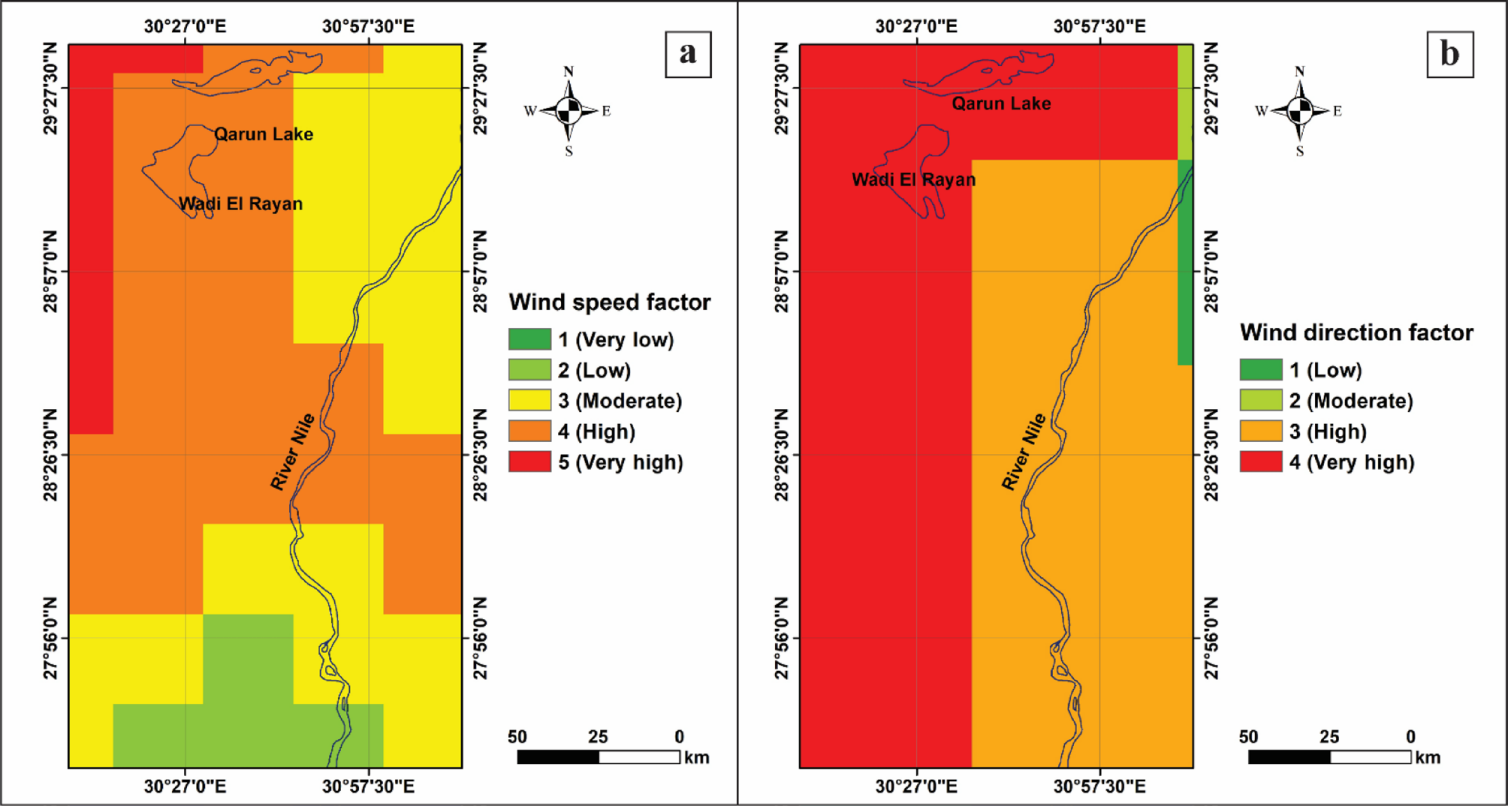
Wind characteristics in the study area (Processed by using ArcGIS 10.5). (**a**) Annual average wind speed classification and (**b**) classification of wind direction factors.

The wind speed factor is crucial to understanding the capacity of winds to erode and transport sand deposits. Furthermore, it assists in determining the initiation, intensity, and pattern of sand movement as it relates to the formation and shape of sand dunes, which are influenced by wind speed and direction^20^. Therefore, analyzing wind speed patterns provides crucial insights into understanding how sand moves, thereby identifying risk zones and developing strategies to mitigate its impact on agriculture. In this study, wind speeds in the study area were categorized into intervals: 1.82–2.82 m/s (1), 2.83–3.4 m/s (2), 3.41–3.88 m/s (3), 3.89–4.36 m/s (4), and 4.37–5.52 m/s (5), as shown in Figs. 2 and 3a. These variations are related to elevation, and extreme temperature differences between day and night can lead to strong winds, which play a significant role in sand transport within the study area. Figures 2 and 3a provide insight into predicting areas most at risk for sand movement, which can inform strategies to mitigate sand encroachment on agricultural land.

#### Wind direction

Wind direction is considered one of the fundamental factors for studying sand encroachment, as it determines the patterns and extent of sand movement. Understanding the prevailing and seasonal wind patterns helps predict which areas are at risk, determine the orientation of sand dunes, and inform the development of effective mitigation strategies. As such, the wind direction factor significantly influences the alignment and type of sand dunes^34^. We created a wind direction tendency map using the Giovanni platform, averaging data from 1947 to 2015. Wind directions were categorized to reflect their impact on sand mobility: 225–315° (1), 180–225° (2), 315–360° (3), and 0–45° (4), as shown in Fig. 3b. These variations in wind direction significantly influence the patterns, intensity, and areas affected by sand encroachment over the study area.

#### Soil characteristics

**Fig. 4:**
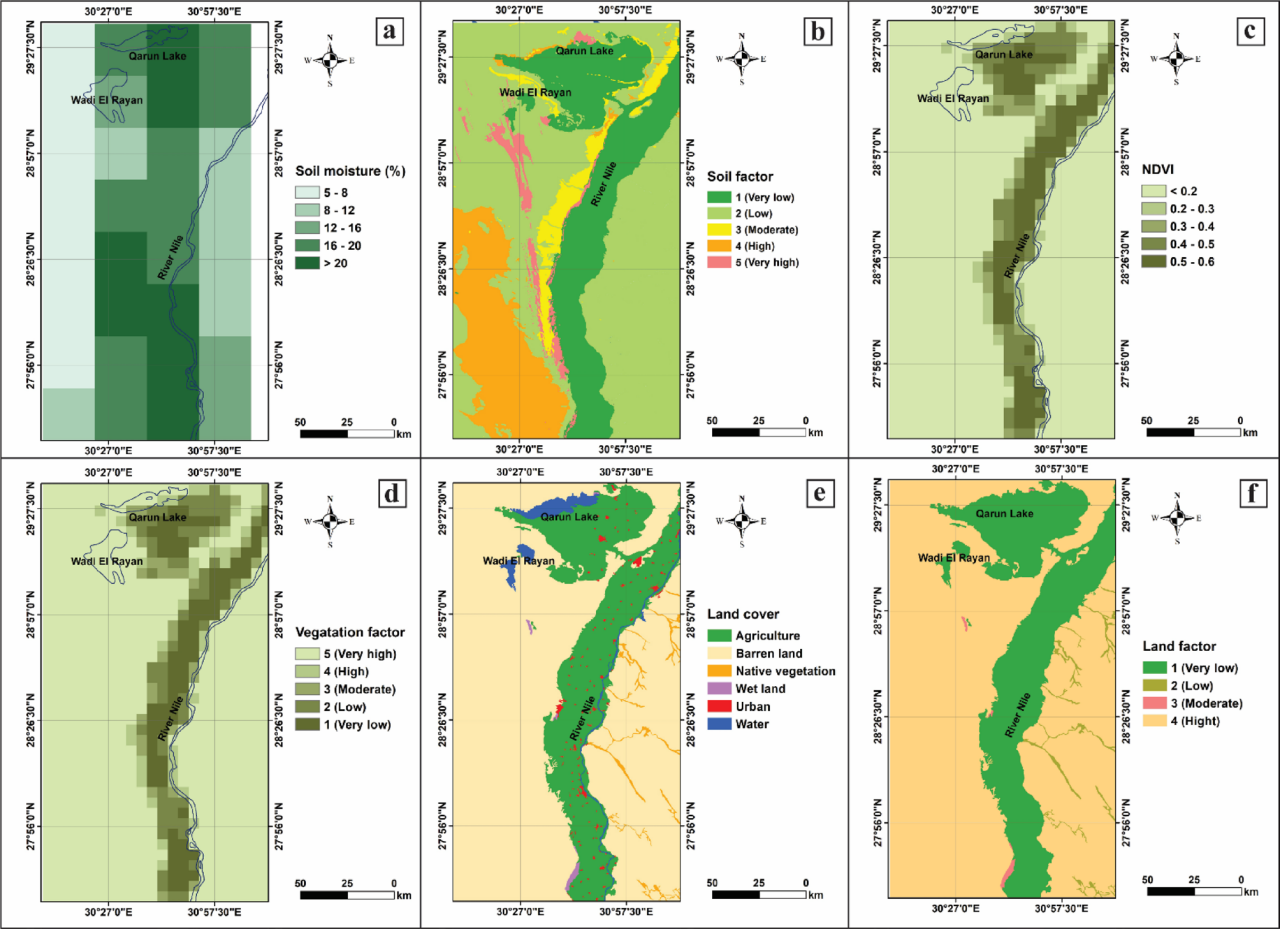
Surface physical properties of the west El Minya area (Processed by using ArcGIS 10.5). (**a**) Soil moisture (ESA CCI SM v03.2), (**b**) Soil factor (SoiFa), (**c**) Vegetation density (ESA CCI Land Cover Africa 2016), (**d**) Vegetation factor classification, (**e**) Land cover (ESA CCI Land Cover 2016), and (**f**) Land factor classifications.

The soil factors governing sand dune dynamics, which incorporate lithology and soil moisture, are crucial in arid climates where soil moisture significantly influences sand mobility. This is because it directly impacts the soil’s susceptibility to wind erosion, its ability to resist sand movement, and the overall stability of the landscape. For instance, loose and dry soils are more susceptible to wind erosion, which accelerates the movement of sand dunes towards agricultural and inhabited areas. Therefore, understanding the role of soil lithology and moisture is essential for predicting when and where sand encroachment is most likely to occur. In our study, we collected soil moisture observations from 1978 to 2015 at a 0.25° spatial resolution. Soil moisture values were reclassified: 5–8% (5), 8–12% (4), 12–16% (3), 16–20% (2), and > 20% (1) (Fig. 4a). Each geological unit was reclassified based on consolidation and resistance to mobility, and a soil cohesiveness factor was calculated by averaging soil moisture and lithology parameters, as shown in Fig. 4b. These classifications of both soil moisture and lithology show the vulnerable zones where sand can easily be eroded and transported and reveal the sources and characteristics of the sand itself.

#### Vegetation cover

Vegetation cover is considered a crucial factor in stabilizing and mitigating the movement of sand dunes. In arid areas such as West El-Minya, the sparse precipitation patterns and residual soil moisture affect such desertic areas^39,40^. Therefore, analyzing the relationship between vegetation coverage and dune dynamics is essential for preventing further encroachment in regions where sand dunes threaten agricultural lands, settlements and infrastructure (Fig. 1c). In our study we classify the vegetation density into five categories: (1) trees and open water; (2) grassland and cropland; (3) aquatic or regularly flooded vegetation; (4) sparse vegetation or shrub-covered land; and (5) bare soil and urban areas (Figs. 4c, d). Figure 4d presents a spatial distribution map of the “vegetation factor” in a geographic region encompassing the River Nile, Qarun Lake, and Wadi El Rayan. We use five classes to represent vegetation factor levels of Very Low, Low, Moderate, High, and Very High. The central corridor along the River Nile shows predominantly darker colors (vegetation factors 1 and 2), which indicate low or very low vegetation levels in most of the area adjacent to the riverbank. Areas farther from the Nile riverbank, notably towards the eastern and western edges, are shaded in lighter greens that reveal higher vegetation factors. The regions surrounding Qarun Lake and Wadi El Rayan are characterized by higher vegetation factors compared to the immediate Nile corridor; however, they still exhibit a mix of low to moderate vegetation. The scale bar indicates coverage of approximately 0 to 50 km, emphasizing that this pattern of low vegetation along the Nile with increasing vegetation away from the river holds throughout the mapped area. In summary, vegetation is lowest directly along the Nile corridor and increases in areas farther from the river, especially toward the map’s eastern and western boundaries (Fig. 4d).

#### Land use

Land use has a direct impact on the dynamics of dune movement, stabilization, and the overall ecosystem balance in the surrounding area. Different land use types, such as agriculture, urban development, grazing, and conservation areas, have varying impacts on the landscape’s ability to resist or succumb to sand encroachment. Decision-makers and planners can develop more targeted strategies for preventing dune migration and mitigating its effects by understanding and classifying the interactions between different land uses and dune systems. In the present study, each land unit was classified based on vulnerability to sand invasion: bare areas (1); vegetation from dense to sparse (2); rivers and channels (3); roads and crops (4); and urban infrastructures (5), as shown in Fig. 4e and f. The Nile River forms the central axis, supporting dense agriculture and urbanization along its course, with almost no agriculture or settlements further away from the river. Consequently, most of the landscape away from direct riverbank access is barren, underscoring the critical dependence on freshwater sources for agriculture and habitation. Therefore, urban areas are relatively small and localized, which reinforces the pattern of settlement and clustering near water resources. As such, this map highlights the strong relationship between water availability, agriculture, and settlement in the region. In addition, it highlights the Nile’s significance in sustaining human activity and vegetation in an otherwise arid landscape (Fig. 4e, f).

#### Surface elevation

**Fig. 5:**
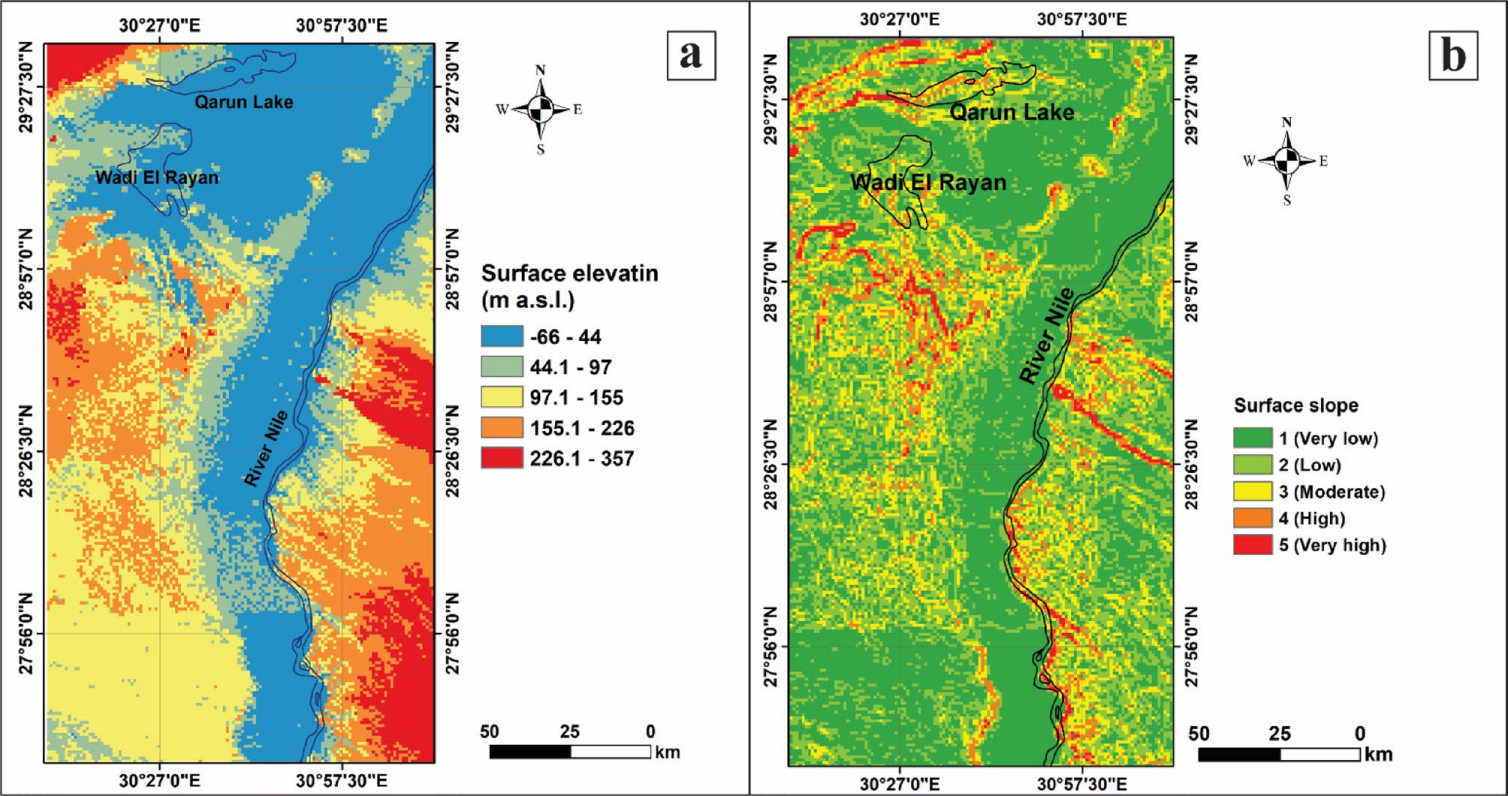
Surface textural properties of the study area (SRTM DEM (30 m)), processed by using ArcGIS 10.5. (**a**) Categories of surface elevation and (**b**) Categories of surface slope.

The elevation affects the speed of sand dunes encroachment upon surrounding areas. The rate of movement varies significantly between low and high dunes or sand formations. Previous studies have shown a relationship between the rate of sand movement and dune altitude^41–43^. It also shows that sand dune activity is more prevalent at higher elevations (Effat et al., 2011). In our study, Surface elevation was classified as −66 to 44 m (1), 44.1 to 97 m (2), 97.1 to 155 m (3), 155.1 to 226 m (4), and 226.1 to 357 m (5), as shown in Fig. 5a. This figure demonstrates that there is a clear topographic gradient where the lowest elevations align tightly with the Nile banks, while elevations increase with distance from the river in both directions. Furthermore, high-elevation areas (orange and red) are separated from the Nile by broad bars (green and yellow), suggesting that substantial escarpments or plateaus border the floodplain. Moreover, Qarun Lake and Wadi El Rayan are situated within the low-lying (blue) terrain, emphasizing their roles as natural depressions. This map highlights the dramatic topographic contrast between the deeply incised Nile valley and the surrounding plateaus, explaining the physical and human geography of the region (Fig. 5a).

#### Surface slope

The slope factor plays a crucial role in studying sand dune encroachment, as the slope or gradient of the terrain and sand dunes affects their movement, accumulation, and stability. Additionally, the slope factor alters the wind interaction with the dune surface, transportation, and the susceptibility of areas to encroachment. As such, the slope is a critical factor that provides insights into sand advancement, with higher slope angles linked to greater vulnerability (Garcia-Mora et al., 2001). In our study, the Surface slope classified categories are: 0–2% (1), 2–5% (2), 5–10% (3), 10–15% (4), and > 15% (5) (Fig. 5b). This map demonstrates that the Nile’s valley floor is nearly continuously on a very low slope, indicating flat terrain optimal for agriculture and human settlement. Furthermore, the high and very high slopes (orange/red) appear as bands or corridors traversing the lowlands and marking the transition to upland terrain or bedrock features. These areas are more liable to runoff and less suitable for extensive agriculture. Moreover, the regions surrounding Qarun Lake and Wadi El Rayan exhibit a mosaic of low to moderate slopes, indicating some elevational relief, but generally not as steep as the highlighted escarpments. On the west bank of the Nile River, numerous moderate- to high-slope patches interrupt the low-lying landscape, revealing geological or topographical variability. This map effectively illustrates the relationship between topography and hydrology in the region, highlighting the dominance of lowland terrain in the Nile floodplain and the significance of elevated slopes as natural boundaries and constraints for land use (Fig. 5b).

#### Sand Dune encroachment vulnerability index (SDEVI)

**Fig. 6:**
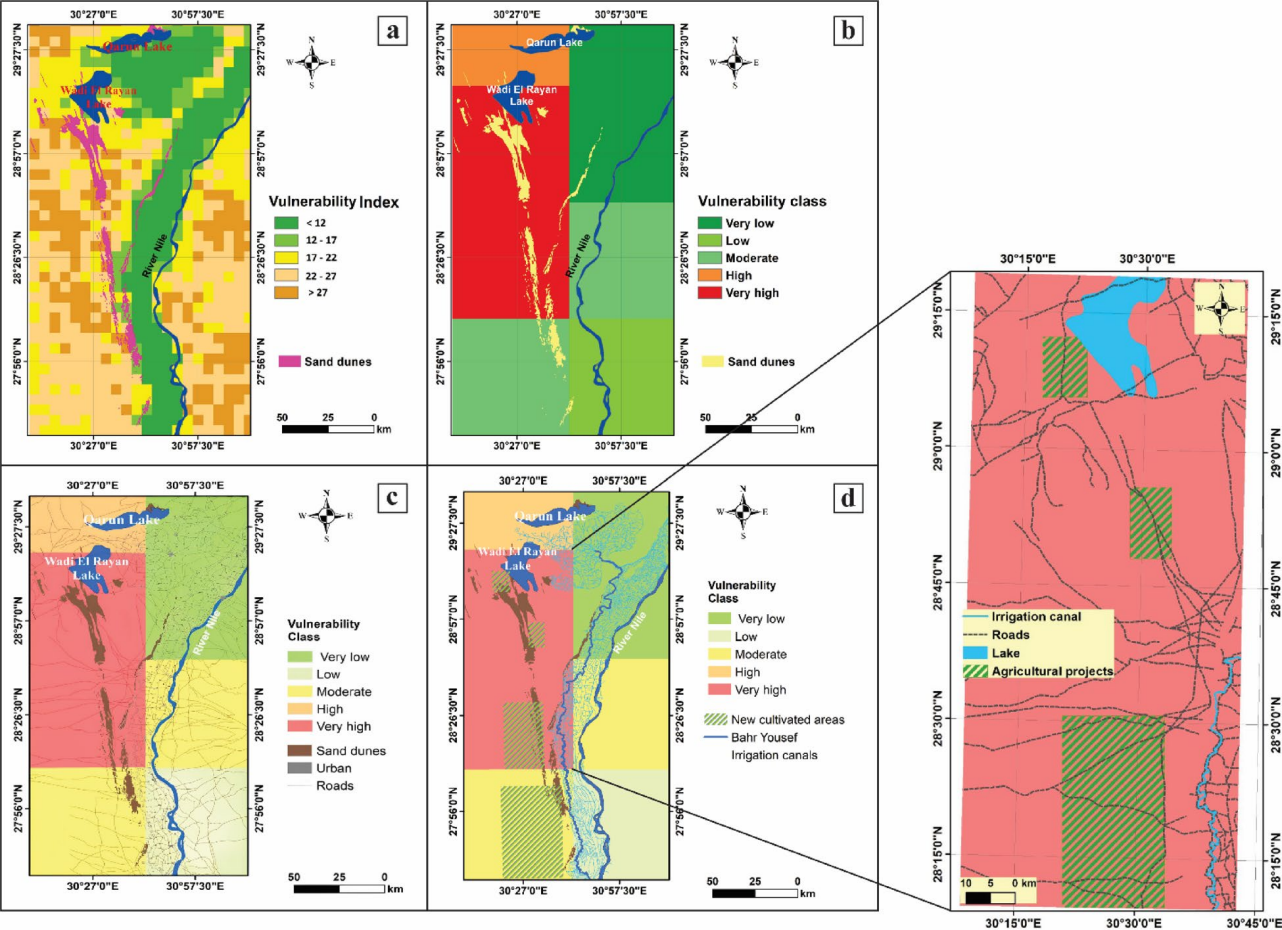
Vulnerability of study area to dune encroachment (Created by using ArcGIS 10.5). (**a**) Sand dune vulnerability index, (**b**) Classification of sand dune vulnerability index (**SDEVI**), (**c**) Urban areas and roads, (**d**) Newly cultivated areas and irrigation canals, and (e) Vulnerability map of the most dangerous area.

The SDEVI incorporates seven variables: wind speed (SpeFa), wind direction (DirFa),Surface elevation (EleiFa), slope (SloFa), land use (LanFa), vegetation (VegFa), and soil cohesion (SoiFa). Vulnerability is classified into five levels: very low (< 12), low (12–17), moderate (17–22), high (22–27), and very high (> 27) (Fig. 6a, b). The vulnerability map shows the lowest vulnerability in the northeastern and southeastern parts, moderate values predominant across the study area, and the highest values in the eastern part (Fig. 6b). Roads in the west of El-Minya governorate near the floodplain, including the Western Desert Road (R1), the internal road west of Baher Youssef (R2), and the internal road east of Baher Youssef and the Nile River (R3), are highly vulnerable to sand dune encroachment (Figs. 1c and 6c and d). The R1 road, connecting Cairo to Upper Egypt, is particularly vulnerable. In El-Minya, the western Baher Youssef area exhibits medium to high vulnerability, whereas the east has medium to low values (Figs. 1c and 6c).

In some areas west of Baher Youssef, sand dunes encroach on the floodplain, affecting historical and cultural artifacts, as well as agricultural land (Fig. 6d). It is hypothesized that the sand dunes have shifted the Bahr-Youssef channel eastward. Monitoring current dune migration rates, sand flux estimates were produced^44^ (Fig. 6c).

Sand dunes accumulating in the Nile floodplain in Middle Egypt (between Samalut and Malawi districts) threaten farmlands and local communities. Local farmers have implemented coping strategies to mitigate the impacts of sand drift, including relocation in some areas^45^. Sand encroachment poses significant threats to development projects, potentially filling canals if not controlled. Barchan and linear dunes, the latter being more hazardous due to their elongation, impact infrastructure by cutting roads and railways, and covering villages, wells, and cultivated lands (Fig. 1c). Controlling linear dunes is challenging due to their oblique growth angle relative to the prevailing NW-SE winds (Fig. 3b).

### Economic implications for the very high vulnerability zone

A detailed spatial analysis was carried out utilizing thematic layers comprising roads, the Bahr Youssef Canal, and both old and new agricultural lands to preliminary quantify the impact of sand dune encroachment across the study area (Fig. 1c). The findings highlight the considerable impacts to several critical infrastructure and agricultural productivity (Fig. 1c; Table 1).

Table 1 presents a preliminary quantitative assessment of the impacts of sand dune encroachment on various infrastructure and agricultural zones and focuses on the Nile riverbanks and adjacent reclaimed lands (Fig. 1c). The table delineates the extent of encroached sand dunes, the length of affected infrastructure, and economic metrics, including the economic impact of encroachment, costs of reclamation, and future risk projections over the next decade.

Our analysis reveals that the Old cultivated area in El-Minya governorate (totalling ~ 176,813 ha; Fig. 1c) experiences sand dune encroachment on 6,562.9 ha, with an estimated economic impact of $52.6 million per year, considering the average economic impact of encroachment of 1 ha is equal to ~$8,011 per year^46^. Approximately 957 hectares are at risk over the next 10 years, with a projected impact of approximately $7.7 million per decade (Table 1). These lands represent the traditional farming regions within the Nile Valley. Furthermore, newly reclaimed lands, located west of the study area, are the most vulnerable, with 26,279.8 ha affected. The economic impact here is substantially higher at $210.5 million per year. The area at risk in the newly reclaimed lands over the next decade spans ~3,041 ha, estimated to be ~ $24.4 million in potential losses over 10 years (Table 1), given the annual sand dune movement rate in the study area is 4.4 m/year. Moreover, the Bahr Youssef Canal, a critical waterway, suffers from encroachment across 5,869 ha, which is directly estimated to impact 174.14 km of its infrastructure. The estimated annual economic impact is $31.3 million. In addition, the road network records the highest length of impacted and at-risk infrastructure (432.94 km) over 12,209.8 ha of sand dune encroachment, which can incurs an annual economic impact of $6.5 million for road clearance from dune encroachment, with several key transport segments severely affected (Fig. 1c; Table 1).

Our study identifies a high vulnerability zone within the area, which poses considerable economic risks and could impact agriculture, Wadi El Rayan Lake, road infrastructure, and the Bahr Youssef irrigation canal (Figs. 1c and 6e). This zone encompasses agricultural land, equivalent to ~42% of the region’s newly established cultivated area, placing it at heightened risk for soil erosion and land degradation. Such degradation could significantly reduce agricultural productivity by 25%, which could economically cost ~ $52 million per year on average^46,47^, driving up land rehabilitation costs and potentially leading to the displacement of farming communities, as well as increased migration in search of a stable livelihood.

Aggregated totals reflect a cumulative sand dune encroachment of 32,842 ha, which accounts for ~ 14% of the total cultivated area of the El-Minya government, which equals ~ 238,204 ha, 607.08 km of infrastructure affected, with total direct economic impact of $300.9 million/year. Therefore, the annual local agriculture GDP will be reduced by 14%. Additionally, the total projected risk to cultivated areas over the next decade is ~ 3,998 ha, with a corresponding long-term economic impact of $32.1 million per year. The latter is a significant amount, as 49% of the local population in the El-Minya governorate works in the agricultural sector^48^. Despite the governorate’s agricultural wealth, almost 60% of El-Minya’s population lives below the poverty line^48^. Many of the impoverished are farmers who suffer from a shortage of subsidized fertilizers and live in an environment where they lack social security and protection.

The above analysis highlights that newly reclaimed agricultural lands are disproportionately affected, with both the highest incidence of damage and significant economic loss, underscoring the need for proactive reclamation and sand dune management strategies.

## Discussion and mitigation strategies

Sand dune encroachment that affects the agricultural lands of the western Nile Riverbanks in El-Minya Governorate, Egypt, provides a clear, data-driven example of the severe impacts of desertification observed across similar areas in North Africa. The severity of these environmental challenges underscores the necessity for the vulnerability assessment approaches applied in our study area and in other sensitive regions of Egypt, such as the Red Sea coast^49^. The analysis presented in Table 1 highlights the magnitude and complexity of these challenges for agriculture, local populations, critical infrastructure, and sustainable development (Fig. 1c) ³.

Our investigation suggests that sand dunes currently encroach upon approximately 50,922 ha in West El-Minya alone, with the most severe losses affecting new cultivation zones (26,280 ha is impacted, Fig. 1d). This mirrors risks faced across Egypt, where a significant area of land are susceptible to advancing dunes, notably in the Western Desert, Sinai and major oases. The continued spread of dunes, wind-driven, threatens the finite supply of fertile land throughout the Nile Valley and beyond, subsequently undermining Egypt’s agricultural stability. For instance, our results reveal that the continued spread of dunes at the rate of 4 m and the associated dust storms in El-Minya puts ~ 400 ha of fertile land at risk annually. Similar large-scale impacts are reflected in Tunisia, Algeria, and Morocco, where significant tracts of productive land are repeatedly destroyed by encroaching sand^5,26^.

Our findings demonstrate substantial impacts on crop production, with the annual agricultural economic loss from encroached areas estimated to be $8,012 per hectar^46^. Hence, the new reclaimed lands are expected to face total economic impacts of $ 210.5 million per year (Table 1). Projections indicate that an additional 3,998 ha will be at risk over the next decade in this region alone. As elsewhere in North Africa, sand burial reduces productivity by 25% per year, ruins irrigation infrastructure, and renders the land less fertile, as evidenced in Algerian oases, where yield losses and direct threats to essential crops, such as date palms and cereals, are now common^4,50^.

Moreover, we quantify the immediate and long-term economic burdens: total estimated losses in West El El-Minya alone reach $300.9 million per year. Such figures illustrate the devastating effects of encroachment on rural economies, which exacerbate poverty and reduce the quality of life throughout the region. These trends are repeated across similar semi-arid areas in North Africa, where sand management, land rehabilitation, and declining agricultural outputs drive economic decline.

Furthermore, infrastructures over 607 km of critical transport and water networks—including major irrigation channels like the Bahr Youssef Canal—are already compromised by advancing sand. Sand-clogged irrigation systems reduce water delivery efficiency, increase evaporation, and restrict both surface and groundwater replenishment. These compounding pressures are repeated in oases and deserts from Egypt to Algeria, where groundwater-dependent communities face falling water tables and mounting scarcity^51^.

The reduction in productive cropland and rising economic losses in West El-Minya threaten local and national food security, exacerbating stress observed on the regional scale. Dropping yields mean greater reliance on costly food imports, which can heighten North Africa’s vulnerability to external shocks. Recurrent sand encroachment also undermines the achievement of the Sustainable Development Goals (SDGs), which impede efforts to combat poverty, maintain food sovereignty, and implement integrated land management strategies.

Beyond agriculture, sand encroachment can disrupt critical infrastructure in Egypt and North Africa, including the oil and gas sector. The findings on transportation corroborate similar financial impacts experienced by energy operations, with millions of dollars spent annually on maintenance, sand removal, and preventing interruptions, such as those observed in Egypt’s Western Desert and Algeria’s major oil field^36^.

The resulting SDEVI map reveals that key transportation routes are highly vulnerable to encroachment by sand dunes. It is hypothesized that historical sand dune migration may have even shifted the Bahr-Youssef channel eastward. The encroachment of sand dunes into the Nile floodplain poses a direct threat to farmlands and local communities, leading to coping strategies such as relocation^45^. The elongation and oblique growth angle of linear dunes make them particularly dangerous and difficult to control.

The economic implications are severe. Our analysis identifies a very high vulnerability zone that poses considerable risks to agriculture and infrastructure. This zone encompasses a significant portion of the region’s cultivated area, placing it at heightened risk for soil erosion and land degradation. Such degradation could significantly reduce agricultural productivity, drive up land rehabilitation costs, and potentially lead to the displacement of farming communities. The aggregated economic impact represents a substantial reduction in the local agricultural GDP, which is particularly damaging in a region where a large percentage of the population works in agriculture and lives below the poverty threshold^2^. The finding that newly reclaimed lands are disproportionately exposed signals an urgent need for proactive reclamation and sand dune management strategies. Further sensitivity analyses are recommended to examine the impact of fluctuations in dune movement rates on better quantifying reclamation costs and their impact on the overall economic figure. This comprehensive strategy enables informed decisions about resource allocation for managing sand encroachment, maximizing the sustainability of agriculture, and the durability of infrastructure.

In summary, mitigating sand dune encroachment in populous western Egypt requires a combination of environmental, technological, and policy interventions. We summarize below the applicable strategies for the expansive western Nile riverbank in Egypt and their effectiveness in stabilizing sand dunes to protect vulnerable areas and foster sustainable land-use practices.

### Sand dunes releveling and planting

**Fig. 7:**
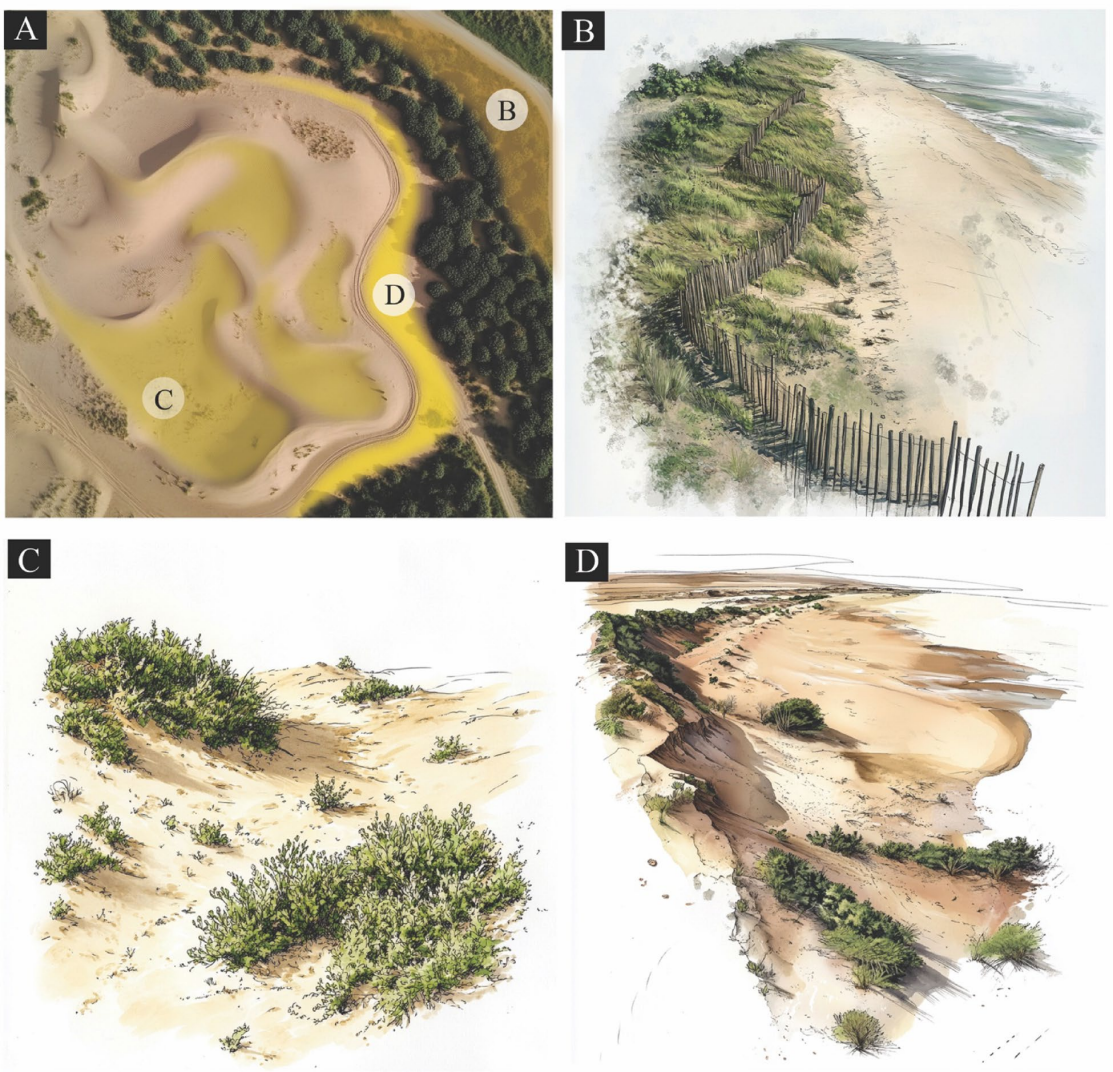
Examples of sand-drift mitigation and dune-stabilization measures. (**A**) Engineered dune field with controlled sand-accumulation zones. (B) Sand fences are installed to reduce wind speed and trap migrating sand. (**C**) Vegetation-based dune stabilization using low, hardy shrubs. and (**D**) Combined structural and vegetative measures to limit erosion and retain sand. The figure was generated by using Midjourney AI (v6) and Adobe Photoshop (2019)..

The process of releveling and planting sand dunes represents the most critical phase in desert reclamation that transforms unstable encroaching sand masses into productive agricultural land (Fig. 7). Based on our data analysis in Tables 1, 50,922 ha of encroached area, and the projected analysis presents an even more urgent figure. Considering the sand dunes’ movement rate at 4.4 m annually and dust storms, an additional 3,998.4 ha face encroachment risk over the next decade, which potentially adds $32.1 million in additional losses under no-mitigation measures. This threat compounds exponentially, making early intervention far more cost-effective than delayed action^38^.

Our study suggests that the releveling operation encompasses ~ 50,922 hectares of encroached land across diverse features, requiring an estimated total cost of ~ $485 million for reclamation (~$9,523/ha)^37^. We recommend that agricultural lands receive high priority for releveling operations, which represent 32,842.7 ha (64.5% of the total affected area) and have combined investment requirements of $312.8 million (Table 1). In particular, the new cultivations affected by dune encroachment account for ~26,280 ha (Fig. 1d) and require an estimated amount of ~ $250 million. Traditional old cultivations, which represent 6,562.9 ha, require an estimated cost of $62.5 million^37^. These areas demonstrate exceptional financial viability with 1.2-year payback periods and annual returns of $263.1 million for mitigated agricultural losses (Table 1).

**Table 1:** Sand Dune Encroachment on Infrastructure and Agricultural Zones and their economic impacts.

Feature	Encroached Sand Dune Area (Hectare)	Affected Infrastructure Length (km)	Economic Impact of the Encroached area ($ million/year)	Cost of Reclamation ($ million)****	Cultivated area (Hectare) at risk for the next 10 years*****	Economic Impact of the Cultivated area at risk ($ million/10years	Remarks
Old Cultivations (176,813 ha)	6,562	–	52.6*	62.5	957	7.7	Farming in Nile Valley
New Cultivations (61,390 ha)	26,280	–	210.5*	250.3	3,041	24.4	Vulnerable reclaimed lands
Bahr Youssef Canal	5,869	174.14	31.3**	55.9	-	-	encroachment zones along canal track
Roads Network	12,210	432.94	6.5***	116.3	-	-	Roads impacted by dune coverage
Total	50,922	607.08	300.9	485	3,998	32.1	

*Considering the annual average agricultural production of 1 Hectare =$ ~8,011 on average^46^.

**Considering the cost of clearing 1 m of drainage from sandstorms is equal to be ~$180^36^.

***Considering the cost of clearing 1 m of a road from sandstorms is equal to be ~$15^36^.

**** Considering the leveling and reclamation cost per one Hectare is equal to $9,523^37^ ($1 ~= 50 EGP). This amount will be paid only once compared to the annual encroachment impact. It is essential to note that leveling and reclaiming these sand dune areas, covering ~ 50,922 ha, will increase the country’s agricultural area and revenue by $300.9 million per year.

***** Considering the sand dunes’ movement rate of 4.4 m/year according to Mohamed^38^ and the associated dust storms.

While infrastructure protection shows lower immediate economic returns, it remains strategically important. For instance, the Bahr Youssef Canal, with 174.14 km of canal infrastructure, is affected by 5,869 ha of sand dunes. Road networks spanning 432.94 km of transportation corridors are impacted by 12,209.8 ha of sand dunes. These areas require specialized approaches of combining mechanical stabilization with infrastructure-specific protection measures.

The economic feasibility analysis of releveling sand dunes and planting reveals a substantial return on investment. The payback period of just 1.2 years for agricultural reclamation makes this mitigation suggestion one of the worthiest public investments. Over a 10-year period, the return on investment can reach 741.2%, generating $3.6 billion in net profits against a total investment requirement of $485 million.

### Landscape-Based stabilization

Nature-based and eco-friendly restoration attempts have gained popularity in waterfront communities, offering both aesthetic value and improved resilience against wind and minor storm events^52–54^. The innovative technique of using colloidal silica-based consolidation to increase the mechanical strength of non-cohesive sediments can reduce dune erosion^55^.

This method enhances the dune system’s resistance and resilience while maintaining its natural characteristics. For some cases, dune stabilization using vegetation can be beneficial; however, it’s crucial to strike a balance, as excessive stabilization can threaten biodiversity. For instance, endemic plant species have been lost due to the creation of a forest succession along the dune boundaries of the Athabasca sand dunes^55^. To address the above, management mitigation is required to sustain or re-establish open sand habitats and the species that depend on them^56^. Biological soil crusts, composed of cyanobacteria, green algae, fungi, mosses, and lichens, play a critical role in stabilizing sand surfaces against wind and water erosion^56^. The latter enhances surface stability and soil fertility, making them key factors in protecting arid and semi-arid ecosystems from desertification^57^.

Moreover, planting trees and shrubs around areas at risk for sand encroachment creates windbreaks that can stabilize the soil and prevent sand movement (Fig. 7). Native species adapted to arid environments, such as Acacia, Tamarisk, and Date Palms, can be used as windbreaks. This method has been successfully applied in arid areas such as Egypt, Algeria, and Morocco. On the other hand, planting hardy grasses and shrubs with extensive root systems helps to tie the soil together and prevent it from being blown away (Fig. 7). In Tunisia, programs involving the planting of halophytes (salt-tolerant plants) have been effective in stabilizing dunes near coastal areas. Additionally, establishing “green belts” or vegetation corridors around vulnerable regions (e.g., agricultural and urban areas) can assist as a natural barrier to dune encroachment (Fig. 7). North African countries such as Algeria have attempted to use this method in several projects, such as the Green Dam Project, which aimed to plant a tree barrier along desert boundaries. Establishing “green belts” or vegetation corridors around vulnerable regions (e.g., agricultural and urban areas) can serve as a natural barrier. This approach, similar to Algeria’s Green Dam Project, should be strategically located based on wind direction (DirFa) and land use (LanFa) factors identified in our SDEVI analysis. For ecologically sensitive dune systems, techniques like colloidal silica-based consolidation can enhance mechanical strength without harming biodiversity^58^, preserving the natural dune dynamics while reducing erosion.

### Hard sand Dune stabilization

In the West El-Minya governorate, field observations and high-resolution satellite imagery indicate that fencing has not yet been widely implemented as a mitigation measure, despite the presence of several high-vulnerability corridors identified in our SDEVI map. These corridors are particularly critical along the Western Desert Road (R1), the Bahr Youssef canal embankments, and the newly reclaimed agricultural blocks. This current lack of physical barriers highlights the urgent need for incorporating well-designed sand fences in future mitigation plans^59,60^.

Given the dominant NW–SE prevailing wind direction (DirFa; Fig. 3b) and the high dune-mobility zones (EleFa and SloFa), the most efficient fencing configuration for the study area would involve serial linear fence arrays placed perpendicular to the prevailing winds^31^ (i.e., oriented NE–SW) to maximize sand trapping efficiency. However, in zones where the topography is more irregular, particularly around active barchan toe areas, zigzag fencing systems are more suitable, as they reduce edge-flow acceleration and enhance sand deposition uniformity^29,31^.

Drawing on successful dune-control projects in similar arid regions of Egypt (e.g., Siwa and El-Kharga), lightweight fences constructed from locally available palm-frond mats (locally known as Jarid) or reed bundles represent the most cost-effective and environmentally compatible options^36^. For high-priority corridors, such as the R1 road, our study recommends budgeting for robust stabilization methods, estimated at approximately $7,910 per kilometer (adjusted for inflation from Ruenkrairergsa, 1982^61^). Based on the observed dune migration rates of ~ 4.4 m/year, the recommended fence spacing in West El-Minya is 8–12 m between successive arrays, ensuring progressive sand accumulation before it reaches agricultural fields.

To further optimize these parameters, recent numerical simulations have proven essential in predicting the ideal spacing, height, and porosity as a function of local environmental conditions. For instance, Computational Fluid Dynamics (CFD) simulations have been used to determine the critical spacing required to minimize wind shear velocity over terrain with sand fences^62^. Integrating such advanced modeling techniques in future site-specific studies could significantly elucidate how sand erosion patterns can be counteracted by the aerodynamic optimization of aeolian fences.

In specific, localized scenarios, the application of soil-binding chemicals or geotextiles can provide temporary stabilization. However, these should be used cautiously due to potential environmental impacts and are best suited for protecting high-value infrastructure where mechanical methods alone are insufficient. Integrating these measures with biological stabilization would provide a comprehensive solution for the high-risk SDEVI zones (> 27)^55,63^.

## Conclusion

Our study provides a comprehensive analysis of the drivers of sand dune encroachment in West El-Minya Governorate, western Egypt, serving as a representative example for other desert urban areas in North Africa that face the same issue. Our results reveal substantial implications that threaten ongoing sustainable agricultural development on the western Nile riverbanks in Egypt due to accelerated sand dune encroachment.

The SDEVI analysis along the study area reveals that the western part is suffering from severe risk, with vulnerability scores above 27 in several critical spots (Fig. 6). This high level of risk is indicated near key infrastructure and farmland, where scores range from high (22–27) to very high (above 27). For example, roads in the western El-Minya governorate, including the Western Desert Road (R1) and smaller roads near Baher Youssef, show some of the highest risk levels (i.e. Figure 1c). These findings emphasize the urgent need to act by boosting vegetation, stabilizing dunes, and improving how the land is managed to protect communities and crops from further sand encroachment. Strong winds blowing mostly from the northwest-southeast at speeds between 4.37 and 5.52 m/s, combined with very low vegetation cover, are considered the main reasons for these high-vulnerability zones. These factors make the area particularly prone to sand dune encroachment.

To address these implications, it is necessary to implement applicable mitigation measures based on the SDEVI findings. Additionally, continuous monitoring and adaptive management strategies should be applied to balance the dynamic nature of sand encroachment and its effects on the western Nile riverbank’s agricultural and infrastructural development.

Our suggested mitigation measures, such as releveling and planting, transform what appears to be an environmental challenge into an exceptional economic opportunity. As the $9,523 per hectare investment generates ~$8,011 per hectare in mitigated annual losses, this creates a 1.2-year payback period, ranking among the most attractive public infrastructure investments available. Furthermore, adaptive green landscape solutions for mitigating sand dune encroachment should focus on maintaining healthy, equilibrated, and dynamic dunes while recovering their natural functioning. The latter requires a quantitative understanding of dune systems and a commitment from stakeholders to implement and integrate management programs for the high-vulnerability zones. By combining nature-based and eco-friendly restoration techniques, we can develop sustainable strategies to address the challenges caused by sand dune encroachment in a changing climate.

Refining the SDEVI factors and exploring additional mitigation strategies to further protect the study area’s sustainable development goals is highly recommended for future work.

Finally, the implications of sand dune encroachment in West El-Minya in Egypt provide a powerful example of the desertification challenges faced by North Africa. The study area’s experience underscores the urgent need for coordinated efforts to address environmental degradation, safeguard agricultural land, and support vulnerable communities. Understanding and addressing the significant challenges in West El-Minya can help policymakers and stakeholders across North Africa develop more effective strategies to combat desertification and promote sustainable development throughout the continent.

## Data Availability

The datasets generated and analyzed during the current study are available from the corresponding author on reasonable request. This includes the wind speed and direction data from the GLDAS Model and MERRA dataset, soil moisture data from the ESA CCI SM v03.2 products, and land cover data from the ESA CCI Land Cover of Africa 2016 map. Additionally, the Digital Elevation Model (DEM) produced by the Shuttle Radar Topography Mission (SRTM) used in this study is publicly accessible. Access to these data sets and materials will be granted in accordance with applicable institutional and ethical guidelines.
